# Fully automated condyle segmentation using 3D convolutional neural networks

**DOI:** 10.1038/s41598-022-24164-y

**Published:** 2022-11-29

**Authors:** Nayansi Jha, Taehun Kim, Sungwon Ham, Seung-Hak Baek, Sang-Jin Sung, Yoon-Ji Kim, Namkug Kim

**Affiliations:** 1 Graduate School of Medicine, University of Ulsan College of Medicine, Seoul, Republic of Korea; 2 Department of Biomedical Engineering, Asan Medical Institute of Convergence Science and Technology, Asan Medical Center, University of Ulsan College of Medicine, Seoul, Republic of Korea; 3 Department of Convergence Medicine, Asan Medical Center, Asan Medical Institute of Convergence Science and Technology, University of Ulsan College of Medicine, Seoul, Republic of Korea; 4 Research Strategy Team, Korea University College of Medicine, Seoul, Republic of Korea; 5 Department of Orthodontics, School of Dentistry, Dental Research Institute, Seoul National University, Seoul, Republic of Korea; 6 Department of Orthodontics, Asan Medical Center, University of Ulsan College of Medicine, Seoul, Republic of Korea

**Keywords:** Health care, Medical research, Signs and symptoms, Mathematics and computing

## Abstract

The aim of this study was to develop an auto-segmentation algorithm for mandibular condyle using the 3D U-Net and perform a stress test to determine the optimal dataset size for achieving clinically acceptable accuracy. 234 cone-beam computed tomography images of mandibular condyles were acquired from 117 subjects from two institutions, which were manually segmented to generate the ground truth. Semantic segmentation was performed using basic 3D U-Net and a cascaded 3D U-Net. A stress test was performed using different sets of condylar images as the training, validation, and test datasets. Relative accuracy was evaluated using dice similarity coefficients (DSCs) and Hausdorff distance (HD). In the five stages, the DSC ranged 0.886–0.922 and 0.912–0.932 for basic 3D U-Net and cascaded 3D U-Net, respectively; the HD ranged 2.557–3.099 and 2.452–2.600 for basic 3D U-Net and cascaded 3D U-Net, respectively. Stage V (largest data from two institutions) exhibited the highest DSC of 0.922 ± 0.021 and 0.932 ± 0.023 for basic 3D U-Net and cascaded 3D U-Net, respectively. Stage IV (200 samples from two institutions) had a lower performance than stage III (162 samples from one institution). Our results show that fully automated segmentation of mandibular condyles is possible using 3D U-Net algorithms, and the segmentation accuracy increases as training data increases.

## Introduction

The mandibular condyle is the growth center of the mandible**.** During development, the vector and magnitude of mandibular growth can be controlled by using forces generated via orthopedic appliances, such as activators, facemasks, and chin cups^1–3^. After the growth is complete, the condylar head undergoes physiologic remodeling in accordance with the functional load placed on the temporomandibular joint (TMJ). Excessive loading due to parafunctions, such as clenching and bruxism, may lead to degenerative changes in the condyles^4^. In patients undergoing orthognathic surgery, changes in the pattern of functional loading may lead to condylar remodeling^5^. Therefore, an accurate assessment of the condylar morphology is necessary to understand the growth modification changes, diagnose TMJ osteoarthritis, and assess skeletal changes following orthodontic and orthognathic treatments.

Quantitative assessment of condylar morphologic changes requires the construction of a 3D model via the segmentation of volumetric images such as computed tomography (CT)^6,7^. Volumetric segmentation can be done manually, semi-automatically, or fully automatically. Manual and semi-automated segmentations are performed by segmenting the object of interest in each 2D slice image followed by the reconstruction of the 3D volume and can be done using medical image analysis software^8–10^. However, manual or semi-automatic segmentation requires extensive training to acquire accurate condyle segmentation because it is difficult to differentiate the condylar head from the glenoid fossa due to the low bone density of the TMJ area and the lack of contrast, especially in low dose CT images such as cone-beam CT (CBCT)^11^.

Deep learning algorithms have been utilized for automated condylar segmentation. Liu et al.^12^, used U-Net, a convolutional neural network, for the initial segmentation to classify CT images into three regions (condyle, glenoid fossa, and background), and then performed the secondary segmentation using the snake algorithm. Kim et al.^13^, used 2D U-Net to directly segment the condyles in axial slice images, and then performed 3D reconstruction. However, annotating 2D slice images of the target 3D volume is tedious and time-consuming. As a result, algorithms such as active learning and self-supervised learning have been applied to reduce the number of training data^14,15^. Instead of annotating 2D images, Çiçek et al.^16^, proposed using the 3D U-Net architecture, which is based on the previous U-Net architecture but replaced the 2D architecture using 3D volumes as input data, and performed 3D convolutions, 3D max pooling, and 3D up-convolutional layers. The major advantage of the 3D U-Net is that it trains considering 3D integrity of anatomy and pathologic lesions, which is impossible in the 2D U-Net, and that it generalizes well^16,17^. Recently, cascaded 3D U-Net using two or more networks has been used to increase the segmentation performance by using the second networks to detect the region of interest (ROI) and focus training on the target region. Liu et al.^18^, compared the performance of cascaded 3D U-Net for brain tumor segmentation in which dice similarity coefficient (DSC) of cascaded U-Net improved by 0.014, 0.052 and 0.033 for whole tumor, tumor core, and enhanced tumor, respectively.

Ham et al.^19^, proposed an automated segmentation of four components—craniofacial hard tissues, maxillary sinus, mandible, and mandibular canals—from facial CBCTs using the 3D U-Net architecture. They used CBCTs from four cases for the segmentation of whole craniofacial hard tissues and CBCTs from twenty cases for automated segmentation of the mandible. However, the mandibular segmentation model targeted the whole mandible, and thus the segmentation results lacked precision in the condylar region for analytic purposes. Therefore, we sought to develop an auto-segmentation algorithm for mandibular condyles using the 3D U-Net and performed a stress test to determine the optimal dataset size for developing the model.

## Methods

### Data collection

1. As this is a retrospective study, informed consent was waived by the ethics committees of Korea University Medical Center (KUMC) (IRB no. 2019AN0213) and Asan Medical Center (AMC) (IRB no. 2019–0927).
2. All methods were carried out in accordance with relevant guidelines and regulations.

### Procedure

**Figure 1:**
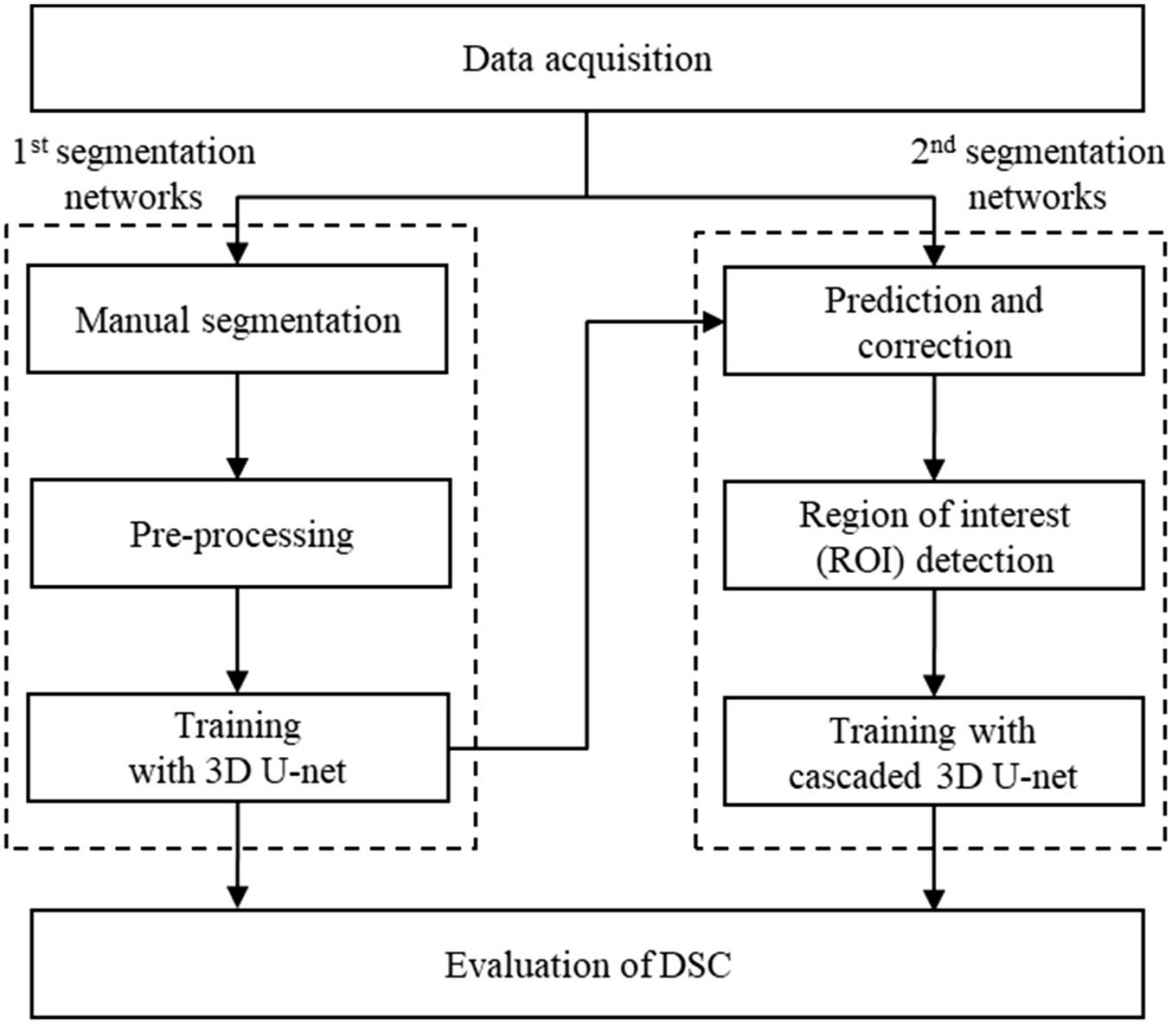
The overall procedures of a basic 3D U-Net and a cascaded 3D U-Net.

The overall study procedure is illustrated in Fig. 1. The acquired CBCT images were manually segmented by experts (TK and NJ) to generate the ground truth, which was further confirmed by a clinician with more than ten years of experience (YJK). After pre-processing, training was carried out using 3D U-Net structures (first segmentation network) and predictions were performed to establish ROIs. The images and labels were cropped, including a margin around the ROI, and training was again performed with a cascaded 3D U-Net (second segmentation network). Accuracy was compared using a basic 3D U-Net and a cascaded 3D U-Net following ROI detection (Fig. 1).

### Dataset

This study was approved by the institutional review boards of Korea University Medical Center (KUMC) (IRB no. 2019AN0213) and Asan Medical Center (AMC) (IRB no. 2019–0927). All subject data were de-identified for the study. We used a CBCT dataset of patients who had visited the department of dentistry and took CBCT from 2018 to 2020. Patients aged 18 to 40 years were included and those who showed osteoarthritic changes in the condyles were excluded. The scanning parameters of CBCT dataset acquired from KUMC and AMC are shown in Table 1. CBCT scans were exported into the Digital Imaging and Communications in Medicine (DICOM) format and de-identified. A total of 234 mandibular condyle images were acquired from 117 subjects. The images of right-side condyles were flipped horizontally so that all images had the same orientation. To determine the optimal number of datasets for auto-segmentation training, a stress test was performed using 80, 120, 162, 200, and 234 sets of condylar images. For stages I, II, and III, images from KUMC were used; for stages IV and V, images from both institutions, KUMC and AMC, were used to evaluate the effect of multi-center data on segmentation accuracy (Table 2).

**Table 1:** Number of data acquired from two centers and scanning parameters (KUMC, Korea University Medical Center; AMC, Asan Medical Center).

Scanning parameter	KUMC	AMC
Manufacturer model	KaVo Dental GmbH	Carestream Health CS 9300
Subject	81	36
FOV (mm)	160.80	167.70
Tube voltage (kV)	120	90
Tube current (mA)	5	5.0
voxel size (mm)	0.30	0.30
Slice thickness (mm)	0.30	0.30

**Table 2:** The data distribution for training, validation, and test in five stages (KUMC, Korea University Medical Center; AMC, Asan Medical Center).

Institutions	Stage I		Stage II		Stage III		Stage IV		Stage V	
	KUMC	AMC	KUMC	AMC	KUMC	AMC	KUMC	AMC	KUMC	AMC
Training	64	–	96	–	130	–	100	60	126	60
Validation	8		12		16		14	6	18	6
Test	8		12		16		14	6	18	6
Total	80	120	162	200	234					

### Manual segmentation and pre-processing

Mandibular condyle segmentation from CBCT scans was performed using the open-source ITK-SNAP software (version 3.4.0; http://www.itksnap.org)^9^. After re-orientation using the orbitale and Frankfort horizontal plane, the condyle head was defined as the ROI. The lower border of the condyle was defined as the horizontal plane that passes through the sigmoid notch. Pre-processing included the following tasks: (1) normalization and (2) image flip and resize.

First, min–max normalization was performed to adjust the contrast on CT images, and it was converted to a value between 0 (minimum) and 1 (maximum) for all features as shown in Eq. (1), with a center of 300 and a width of 2000,

1 $$x_{new} = \frac{{x_{i} - x_{min} }}{{x_{max} - x_{min} }}$$

2 $$x_{min} = \frac{WL - WW}{2}$$

3 $$x_{max} = \frac{WL + WW}{2}$$

where $x_{i}$ and $x_{new}$ are the original and new images, respectively [21]. The window level, often referred to as the window center, is the midpoint of the range of the CT number displayed; the window width is the measure of the range of CT numbers contained in an image.

**Figure 2:**
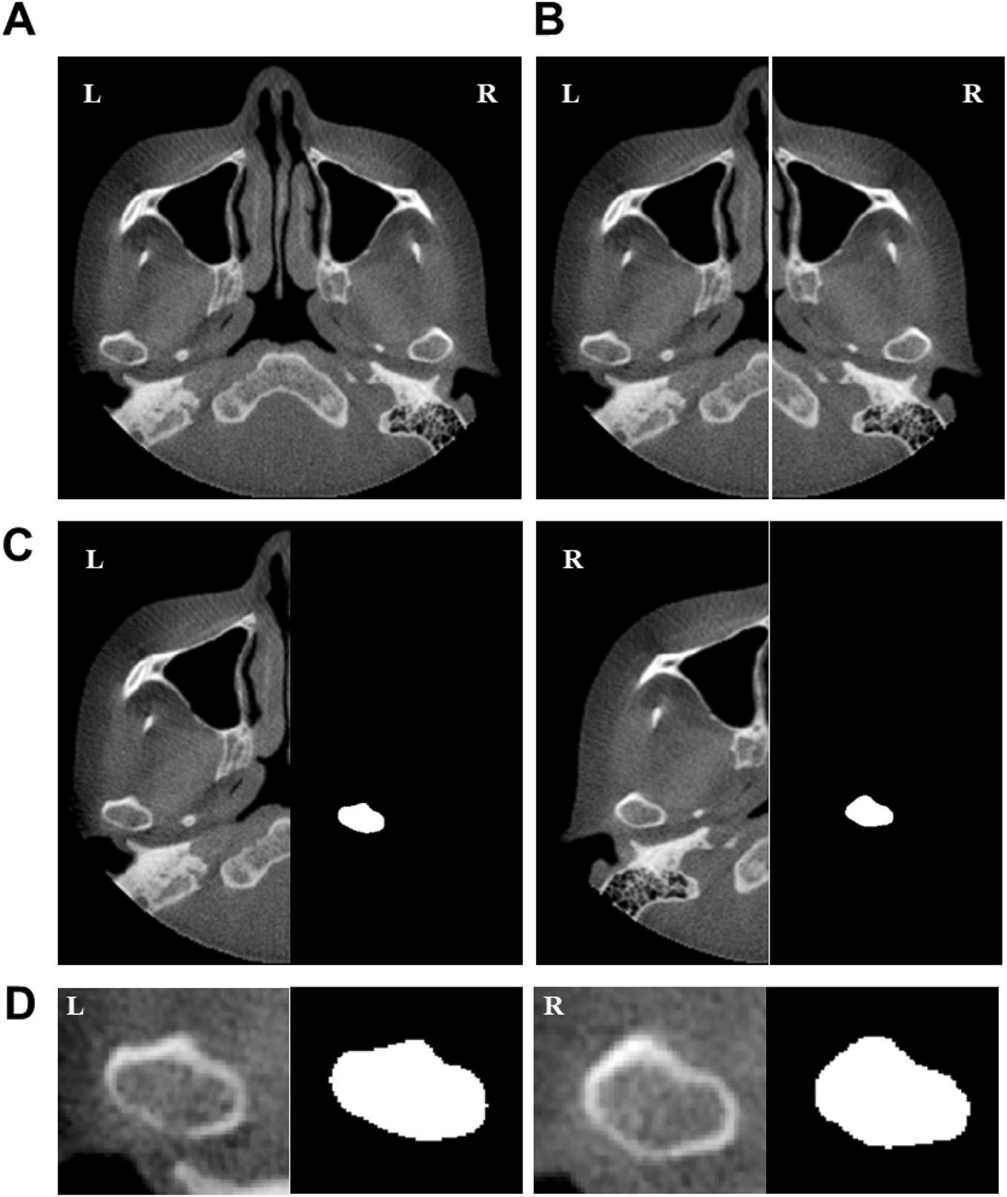
Pre-processing procedure for basic 3D U-Net and cascaded 3D U-Net: (**A**) normalization of the image, (**B**) division into right and left sides, (**C**) left side remained untransformed while right images were flipped horizontally, and (**D**) cropping of the image, including the margin.

Second, since each subject had two mandibular condyles (Fig. 2A), separating the condyles doubled the dataset (Fig. 2B). After dividing the right and left sides in the axial view, the left images were preserved, and the right images were horizontally flipped (Fig. 2C). Images in the first segmentation network were resized to an FOV of 128 × 256 × 192 (Fig. 2C), and images in the second segmentation network were resized to an FOV of 80 × 80 × 80 (Fig. 2D).

### Cascaded 3D U-Net for automated mandibular condyle segmentation

**Figure 3:**
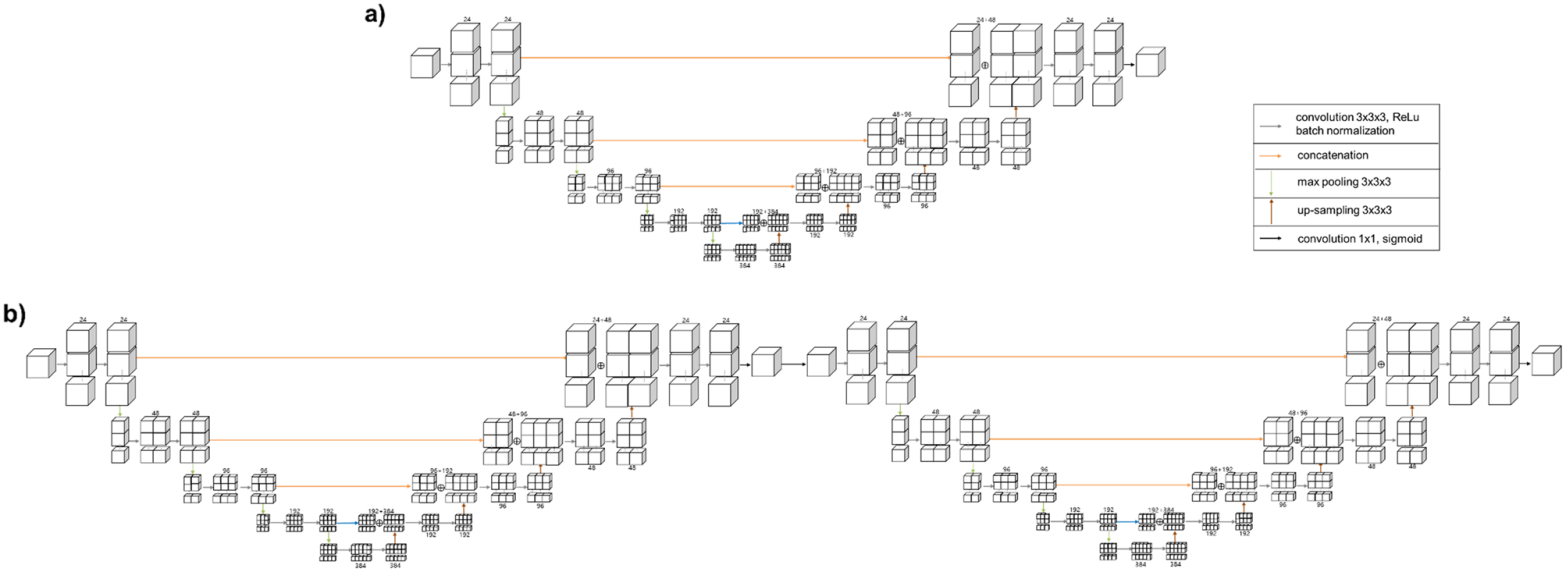
The architecture of (**a**) a basic 3D U-Net and (**b**) a cascaded 3D U-Net.

The process for automated cascaded 3D U-Net consisted of the first (basic 3D U-Net) and second (cascaded 3D U-Net) segmentation networks (Fig. 1). The first segmentation network was used to segment the condyles using a 3D U-Net architecture after pre-processing (Fig. 3a). The second segmentation network was performed after detecting the ROIs (mandibular condyle), including the margin, by using the first segmentation network (Fig. 3b). The same 3D U-Net models were used for the first and second segmentation networks. The margins were set to the maximum (max) and minimum (min) values of the x, y, and z axes for the predicted mandibular condyle region and expanded to an empirically determined value of five pixels. After cropping the medical images and labels to the defined margin region, input datasets were acquired and again trained using 3D U-Net architecture. Finally, the predicted labels of the mandibular condyle were obtained via cascaded 3D U-Net. In 3D U-Net architecture, the left side reduces the number of dimensions whereas the right side extends the original number of dimensions with concatenation, which leads to better segmentation by avoiding the loss of information. The 3D U-Net architecture consisted of 3D convolution, batch normalization, Rectified Linear Unit, up and down sampling, and a 3D max pooling layer (3 × 3 × 3). Training was performed with one batch size. The average dice coefficient loss was used as a training loss. For training, a graphical processing unit of NVIDIA TITAN RTX with available memory of 24,220 MiB was used. The training module was executed in Keras 2.3.0 and Tensorflow 1.15.0 backend.

### Evaluation

DSC was used to investigate the performance of the basic 3D U-Net and the cascaded 3D U-Net. The DSC had a range from 0 to 1, where 0 means no superposition between the volumes and 1 means a perfect superposition between the volumes. DSC was calculated according to Eq. (4),

4 $$DSC \left( {V_{GT} ,V_{Pred} } \right) = \frac{{2\left| {V_{GT} \cap V_{Pred} } \right|}}{{\left| {V_{GT} } \right| + \left| {V_{Pred} } \right|}}$$

where $V_{GT}$ and $V_{Pred}$ were the volume of ground truth and predicted label, respectively, using basic 3D U-Net and the cascaded 3D U-Net.

The Hausdorff distance (HD) was evaluated to compare between ground truth and the predicted labels obtained by the basic 3D U-Net and the cascaded 3D U-Net. HD was defined as an Eq. (5) with sets.$A = \left\{ {a_{1} , a_{2} , a_{3} , \ldots ,a_{n} } \right\}$ and $B = \left\{ {b_{1} , b_{2} , b_{3} , \ldots ,b_{n} } \right\}$

5 $$HD \left( {A,B} \right) = \max \left( {\mathop {max }\limits_{a \in A} \mathop {\min }\limits_{b \in B} A - B, \mathop {max }\limits_{a \in B} \mathop {\min }\limits_{b \in A} B - A} \right)$$

Where $\left\| {\; \cdot \;} \right\|$ is the Euclidean distance, or the distance between point *A* of the ground truth and point *B* of the predicted labels. To compare the difference between basic 3D U-Net and cascaded 3D U-Net, paired *t*-test was used to compare significant differences using IBM SPSS Statistics for Windows (Version 20.0, IBM Corp., Armonk, NY, USA). In addition, the time efficiency was evaluated between the manual and corrected segmentation based on the predicted labels with basic 3D and cascaded 3D U-Nets.

## Results

### Comparison between basic 3D U-Net and cascaded 3D U-Net

Table 3 shows the average and standard deviation (SD) of the DSCs and HDs for each stage. When the use of basic 3D U-Net and cascaded 3D U-Net for all stages were compared, the DSC was higher.

**Table 3:** Dice similarity coefficient and Hausdorff distance for each stage with 3D U-Net only and cascaded 3D U-Net (DSC, Dice similarity coefficient; HD, Hausdorff distance).

Metric	Model	Stage I		Stage II		Stage III		Stage IV		Stage V
DSC	Basic 3D U-Net	0.886 ± 0.034		0.903 ± 0.039		0.910 ± 0.029		0.909 ± 0.026		0.922 ± 0.021
	Cascaded 3D U-Net	0.912 ± 0.043		0.921 ± 0.035		0.928 ± 0.038		0.916 ± 0.032		0.932 ± 0.023
	*P*	0.245		< 0.05		< 0.001		0.120		< 0.01
HD	Basic 3D U-Net	3.099 ± 1.218		2.882 ± 1.061		2.884 ± 0.992		2.491 ± 0.319		2.557 ± 0.250
	Cascaded 3D U-Net	2.494 ± 0.652		2.518 ± 0.460		2.600 ± 0.726		2.538 ± 0.395		2.452 ± 0.332
	*P*	< 0.05		0.081		0.141		0.985		< 0.001

when using the cascaded 3D U-Net than basic 3D U-Net; the DSCs were statistically significant except for stages I and IV (Table 3). Supplementary Table S1 lists the DSC values of all test data.

The HD was higher when using the basic 3D U-Net than the cascaded 3D U-Net except for stage IV. The difference in HDs were statistically significant except for stages II and IV (Table 3). The HDs of the test dataset in all stages were presented in Supplementary Table S2. Table 4 shows the time spent for the manual and corrected segmentation based on the predicted labels obtained with basic 3D U-Net and cascaded 3D U-Net. Mean time for manual segmentation was 14.75 ± 3.63 min, mean time for segmentation using basic 3D U-Net and cascaded 3D U-Net was 4.13 ± 1.94 min and 2.31 ± 1.54 min, respectively.

**Table 4:** Segmentation time for manual, basic 3D U-Net, and cascaded 3D U-Net in Stage V.

Patient No	Manual (min)		Basic 3D U-Net (min)		Cascaded 3D U-Net (min)	
	R	L	R	L	R	L
1	23.08	16.43	5.58	3.75	6.80	6.28
2	14.08	9.83	3.80	6.77	0.73	3.75
3	13.88	10.82	2.98	3.13	1.53	3.05
4	13.08	10.22	1.92	5.93	1.06	2.22
5	17.98	19.87	1.63	4.05	0.45	1.77
6	13.60	10.40	1.1	2.97	1.53	3.13
7	14.36	12.92	2.67	2.75	0.59	1.52
8	15.95	16.90	2.75	4.70	1.66	4.25
9	14.40	14.23	6.50	1.07	0.48	1.75
10	16.92	9.38	6.93	5.68	3.73	2.52
11	18.52	20.28	6.13	7.35	2.77	5.33
12	14.25	14.23	3.87	4.80	1.23	1.73
Mean ± SD	15.98 ± 2.98	13.51 ± 3.93	3.823 ± 2.10	4.412 ± 1.82	1.432 ± 1.01	3.11 ± 1.53

### Stress test results

Among the stages that used a dataset obtained from a single institution, stage III using the 162-sample dataset offered the greatest DSC values of 0.910 ± 0.029 and 0.928 ± 0.038 for basic 3D U-Net and cascaded 3D U-Net, respectively. For the stages that used datasets from two institutions, stage V (234 samples) was found to have a higher DSC than stage IV (200 samples) (Table 3). In addition, the DSC of the cascaded 3D U-Net in stage I (80 samples) was not significantly different from that of the basic 3D U-Net in stage III (162 samples) (*P* = 0.81).

## Discussion

In this study, we examined the potential utility of a cascaded 3D U-Net architecture, a fully automated segmentation model for mandibular condyle with higher accuracy than the comparable non-cascaded architecture. Condyles were distinguished based upon semantic segmentation determined by using the basic 3D U-Net, and then the images were cropped including a margin around the condylar head. As a result, the networks were able to improve their segmentation performance by utilizing a cascaded 3D U-Net. A systematic stress test was performed to determine the optimal number of training data for clinically acceptable accuracy.

**Figure 4:**
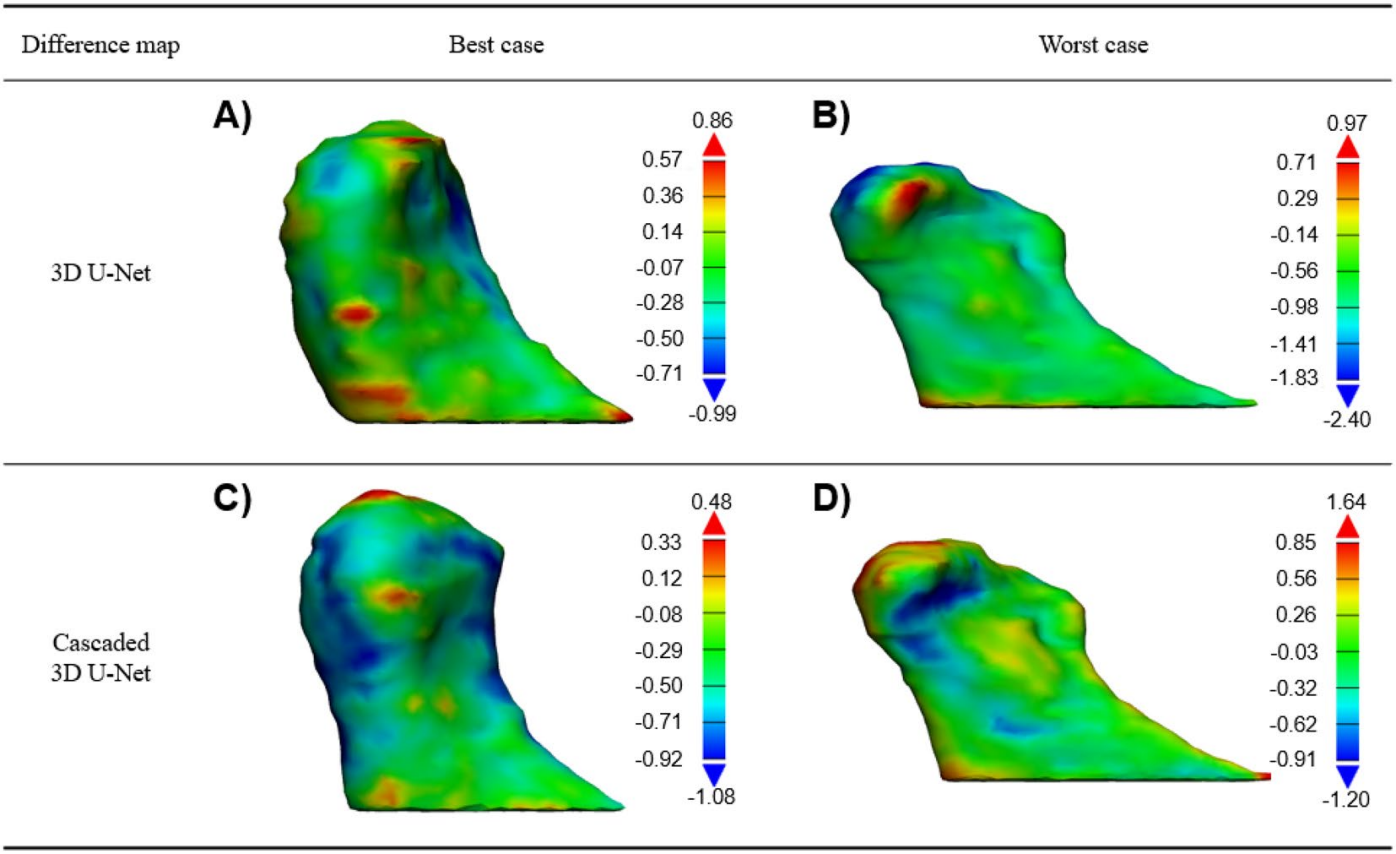
Difference map between ground truth and prediction in basic 3D U-Net and cascaded 3D. U-Net. (**A**) Best case (error range, − 0.99–0.86 mm) and (**B**) worst case (error range, − 2.40–0.97 mm) of basic 3D U-Net. (**C**) Best case (error range, − 0.48–1.08 mm) and (**D**) worst case (error range − 1.20–1.64 mm) of cascaded 3D U-Net.

The mean DSC of the cascaded 3D U-Net was higher than that of the basic 3D U-Net in all stages of test datasets, indicating a higher performance of the cascaded model that includes ROI detection prior to segmentation. In addition, stage I utilizing 80 samples and cascaded 3D U-Net exhibited a similar accuracy to stage III utilizing 162 samples and the basic 3D U-Net (Table 3). However, basic 3D U-Net showed a greater improvement in DSCs and HDs from stages I to V than the cascaded 3D U-Net. This could be explained as that the basic 3D U-Net uses the whole image as input data containing more anatomical information adjacent to the target, whereas the cascaded 3D U-Net uses cropped images of the condyle only. Figure 4 shows the best and worst cases for a difference map between the ground truth and prediction with basic 3D U-Net and cascaded 3D U-Net in stage V. The positive and negative areas depict over-segmentation and under-segmentation, respectively. The best cases of basic 3D U-Net and cascaded 3D U-Net showed a DSC of 0.960 (error range, − 0.99–0.86 mm) and 0.961 (error range, − 0.48–1.08 mm), respectively (Fig. 4A,C); the worst cases of basic 3D U-Net and cascaded 3D U-Net showed a DSC of 0.864 (error range, − 2.40–0.97 mm) and 0.890 (error range − 1.20–1.64 mm), respectively (Fig. 4B,D, Supplementary Table S1). The segmentation time of basic 3D U-Net and cascaded 3D U-Net was significantly reduced by 10.62 min and 12.45 min, respectively, compared to the manual segmentation. In some cases, the segmentation that used basic 3D U-Net was shown to have a higher accuracy than cascaded 3D U-Net (Supplementary Table S1). The reason may be that whereas basic 3D U-Net was trained using full images that included adjacent anatomical structures, the cascaded 3D U-Net was trained using cropped images of the condylar head. Thus, the cascaded 3D U-Net may not readily distinguish the adjacent structures such as maxilla and temporal bone and result in over-segmentation.

The stress test showed that the segmentation performance increased as the dataset size increased for both basic and cascaded 3D U-Net. To compare the segmentation accuracy of single and multi-center data, data from KUMC were allocated for stages I, II, and III, and data from KUMC and AMC were used in stages IV and V. The DSC of stage IV was lower than that in stage III that used 162 samples from a single institution (KUMC). Although the performance of stage III was superior to that of stage IV, algorithms developed in stages IV and V would be more generalizable. In stage V, 34 samples were added from the two institutions from stage IV and exhibited the best performance among all stages. The stress test of HD was excluded because it may contain noise from areas other than the condyles.

There are several limitations to this study. Our study only included the condyles with normal morphologies because arthritic condyles have various morphologies such as flattening, osteophytes, and irregular borders, which is more challenging. These condyles often do not have a cortical bone lining in the images which would require additional training. Therefore, a further study on segmentation of arthritic condyles is warranted. The cascaded 3D U-Net may be useful in segmenting the abnormal condyles by locating the condylar head from the surrounding structures. This will allow diagnosis of TMJ arthritis based on 3D images rather than 2D slices. Also, as we developed the automated segmentation algorithms using datasets obtained from two institutions, future studies should include images from various CBCT machines, which will increase the generalizability of the segmentation algorithms. Lastly, even though the test dataset showed a high mean DSC, the distance map between the ground truth and prediction showed errors as high as 3 mm in some of the segmentations, which may be clinically significant. Therefore, further studies are needed to decrease the error margins.

## Conclusion

Fully automated segmentation of mandibular condyles was possible using 3D U-Net algorithms. Cascaded 3D U-Net architecture showed a higher performance than basic 3D U-Net. Segmentation accuracy increased as the number of training data increased for both cascaded 3D U-Net and basic 3D U-Net. Although the mean accuracy of segmentation was high, the error range may be clinically significant and requires further refinement.

## Supplementary Information

Supplementary Information.

## Data Availability

All data generated or analyzed during this study are included in this published article (and its Supplementary Information files).
