# Supplementary material for: Fully automated condyle segmentation using 3D convolutional neural networks
Source: Sci Rep. 2022 Nov 29;12:20590. doi: 10.1038/s41598-022-24164-y (PMC9709043; doi:10.1038/s41598-022-24164-y)
Supplement: Supplementary file 1 — Supplementary Information. [file 41598_2022_24164_MOESM1_ESM.pdf]

Fully Automated Condyle Segmentation using 3D Convolutional Neural Networks

Nayansi Jha[a†], Taehun Kim[b,c†], Sungwon Ham[d], Seung-Hak Baek[e], Sang-Jin Sung[f], Yoon-Ji Kim[f*], Namkug Kim[c*]

[a]Graduate School of Medicine, University of Ulsan College of Medicine, Seoul, Republic of Korea

[b]Department of Biomedical Engineering, Asan Medical Institute of Convergence Science and Technology, Asan Medical Center, University of Ulsan College of Medicine, Seoul, Republic of Korea

[c]Department of Convergence Medicine, Asan Medical Institute of Convergence Science and Technology, Asan Medical Center, University of Ulsan College of Medicine, Seoul, Republic of Korea

[d] Research Strategy Team, Korea University College of Medicine, Seoul, Republic of Korea

[e]Department of Orthodontics, School of Dentistry, Dental Research Institute,
Seoul National University, Seoul, Republic of Korea

[f]Department of Orthodontics, Asan Medical Center, University of Ulsan College of Medicine, Seoul, Korea

[†]Nayansi Jha and Taehun Kim contributed equally as first authors to this study.

[*]Yoon-Ji Kim and Namkug Kim contributed equally as corresponding authors to this study.

[*]**Corresponding Authors**

Yoon-Ji Kim

Department of Orthodontics

Asan Medical Center, University of Ulsan College of Medicine

88, Olympic-ro 43-gil, Songpa-gu, Seoul, 05505, Republic of Korea

Tel +82-2-3010-3845, Fax: +82-2-3010-6967

E-mail: yn0331@ulsan.ac.kr

ORCiD number: 0000-0002-7030-569X

Namkug Kim

Department of Convergence Medicine

Asan Medical Institute of Convergence Science and Technology

Asan Medical Center, University of Ulsan College of Medicine

88, Olympic-ro 43-gil, Songpa-gu, Seoul, 05505, Republic of Korea

Tel: +82-2-3010-6573, Fax: +82-2-3017-4282

E-mail: namkugkim@gmail.com

Keywords: mandibular condyle, cone-beam computed tomography, convolutional neural networks, image processing, segmentation, temporomandibular joint, stress test

Supplementary Table S1. Dice similarity coefficient of 12 subjects (24 test or tuning sample datasets) for each of five stages using as derived utilizing basic 3D U-Net and cascaded 3D U-Net.

Dataset	Subject No.	Stage 1				Stage 2				Stage 3				Stage 4				Stage 5			
		Basic 3D U-Net		Cascaded 3D U-Net		Basic 3D U-Net		Cascaded 3D U-Net		Basic 3D U-Net		Cascaded 3D U-Net		Basic 3D U-Net		Cascaded 3D U-Net		Basic 3D U-Net		Cascaded 3D U-Net	
		L	R	L	R	L	R	L	R	L	R	L	R	L	R	L	R	L	R	L	R
KUMC	1	0.904	0.871	0.915	0.818	0.908	0.842	0.920	0.827	0.911	0.842	0.923	0.796	0.912	0.914	0.927	0.890	0.921	0.916	0.941	0.918
	2	0.917	0.915	0.956	0.944	0.936	0.930	0.957	0.940	0.926	0.940	0.955	0.954	0.925	0.934	0.953	0.958	0.930	0.937	0.961	0.960
	3	0.821	0.854	0.900	0.920	0.823	0.886	0.910	0.922	0.853	0.895	0.928	0.917	0.822	0.874	0.870	0.935	0.864	0.888	0.890	0.922
	4	0.899	0.909	0.905	0.938	0.949	0.941	0.909	0.951	0.924	0.935	0.926	0.955	0.924	0.936	0.859	0.953	0.935	0.945	0.939	0.958
	5	-	-	-	-	0.919	0.899	0.939	0.899	0.912	0.890	0.937	0.905	0.929	0.934	0.951	0.944	0.932	0.929	0.952	0.952
	6	-	-	-	-	0.895	0.907	0.935	0.944	0.911	0.910	0.941	0.935	0.905	0.901	0.930	0.941	0.901	0.924	0.938	0.933
	7	-	-	-	-	-	-	-	-	-	-	-	-	0.924	0.901	0.874	0.913	0.937	0.920	0.898	0.925
	8	-	-	-	-	-	-	-	-	0.924	0.927	0.939	0.937	-	-	-	-	0.935	0.933	0.942	0.949
	9	-	-	-	-	-	-	-	-	0.929	0.941	0.953	0.948	-	-	-	-	0.931	0.960	0.951	0.946
AMC	10	-	-	-	-	-	-	-	-	-	-	-	-	0.884	0.911	0.887	0.867	0.883	0.901	0.896	0.894
	11	-	-	-	-	-	-	-	-	-	-	-	-	0.896	0.925	0.925	0.890	0.906	0.927	0.919	0.890
	12	-	-	-	-	-	-	-	-	-	-	-	-	0.918	0.912	0.935	0.915	0.931	0.932	0.946	0.940
Mean ± SD		0.886 ± 0.034		0.912 ± 0.043		0.903 ± 0.039		0.921 ± 0.035		0.910 ± 0.029		0.928 ± 0.038		0.909 ± 0.026		0.916 ± 0.032		0.922 ± 0.021		0.932 ± 0.023	

KUMC, Korea University Medicine; AMC, Asan Medical Center

Supplementary Table S2. Hausdorff distance of 12 subjects (24 test or tuning sample datasets) for each of five stages using as derived utilizing basic 3D U-Net and cascaded 3D U-Net.

Dataset	Subject No.	Stage 1				Stage 2				Stage 3				Stage 4				Stage 5			
		Basic 3D U-Net		Cascaded 3D U-Net		Basic 3D U-Net		Cascaded 3D U-Net		Basic 3D U-Net		Cascaded 3D U-Net		Basic 3D U-Net		Cascaded 3D U-Net		Basic 3D U-Net		Cascaded 3D U-Net	
		L	R	L	R	L	R	L	R	L	R	L	R	L	R	L	R	L	R	L	R
KUMC	1	0.904	0.871	0.915	0.818	0.908	0.842	0.920	0.827	0.911	0.842	0.923	0.796	0.912	0.914	0.927	0.890	0.921	0.916	0.941	0.918
	2	0.917	0.915	0.956	0.944	0.936	0.930	0.957	0.940	0.926	0.940	0.955	0.954	0.925	0.934	0.953	0.958	0.930	0.937	0.961	0.960
	3	0.821	0.854	0.900	0.920	0.823	0.886	0.910	0.922	0.853	0.895	0.928	0.917	0.822	0.874	0.870	0.935	0.864	0.888	0.890	0.922
	4	0.899	0.909	0.905	0.938	0.949	0.941	0.909	0.951	0.924	0.935	0.926	0.955	0.924	0.936	0.859	0.953	0.935	0.945	0.939	0.958
	5	-	-	-	-	0.919	0.899	0.939	0.899	0.912	0.890	0.937	0.905	0.929	0.934	0.951	0.944	0.932	0.929	0.952	0.952
	6	-	-	-	-	0.895	0.907	0.935	0.944	0.911	0.910	0.941	0.935	0.905	0.901	0.930	0.941	0.901	0.924	0.938	0.933
	7	-	-	-	-	-	-	-	-	-	-	-	-	0.924	0.901	0.874	0.913	0.937	0.920	0.898	0.925
	8	-	-	-	-	-	-	-	-	0.924	0.927	0.939	0.937	-	-	-	-	0.935	0.933	0.942	0.949
	9	-	-	-	-	-	-	-	-	0.929	0.941	0.953	0.948	-	-	-	-	0.931	0.960	0.951	0.946
AMC	10	-	-	-	-	-	-	-	-	-	-	-	-	0.884	0.911	0.887	0.867	0.883	0.901	0.896	0.894
	11	-	-	-	-	-	-	-	-	-	-	-	-	0.896	0.925	0.925	0.890	0.906	0.927	0.919	0.890
	12	-	-	-	-	-	-	-	-	-	-	-	-	0.918	0.912	0.935	0.915	0.931	0.932	0.946	0.940
Mean ± SD		0.886 ± 0.034		0.912 ± 0.043		0.903 ± 0.039		0.921 ± 0.035		0.910 ± 0.029		0.928 ± 0.038		0.909 ± 0.026		0.916 ± 0.032		0.922 ± 0.021		0.932 ± 0.023	

KUMC, Korea University Medicine; AMC, Asan Medical Center
